# Synovial Mesenchymal Stem Cells Promote Meniscus Regeneration Augmented by an Autologous Achilles Tendon Graft in a Rat Partial Meniscus Defect Model

**DOI:** 10.1002/stem.2030

**Published:** 2015-05-21

**Authors:** Nobutake Ozeki, Takeshi Muneta, Seiya Matsuta, Hideyuki Koga, Yusuke Nakagawa, Mitsuru Mizuno, Kunikazu Tsuji, Yo Mabuchi, Chihiro Akazawa, Eiji Kobayashi, Tomoyuki Saito, Ichiro Sekiya

**Affiliations:** a Department of Joint Surgery and Sports medicine; b Department of Orthopaedic Surgery, Yokohama City UniversityYokohama, Japan; c Center for Stem Cell and Regenerative Medicine, Tokyo Medical and Dental UniversityTokyo, Japan; d Department of Cartilage Regeneration, Graduate School of Medicine; e Department of Biochemistry and Biophysics, Graduate School of Health care Science, Tokyo Medical and Dental UniversityTokyo, Japan; f Department of Organ Fabrication, Keio University School of MedicineTokyo, Japan

**Keywords:** Stem cell transplantation, Mesenchymal stem cells, Arthritis, Cellular therapy

## Abstract

Although meniscus defects and degeneration are strongly correlated with the later development of osteoarthritis, the promise of regenerative medicine strategies is to prevent and/or delay the disease's progression. Meniscal reconstruction has been shown in animal models with tendon grafting and transplantation of mesenchymal stem cells (MSCs); however, these procedures have not shown the same efficacy in clinical studies. Here, our aim was to investigate the ability of tendon grafts pretreated with exogenous synovial-derived MSCs to prevent cartilage degeneration in a rat partial meniscus defect model. We removed the anterior half of the medial meniscus and grafted autologous Achilles tendons with or without a 10-minute pretreatment of the tendon with synovial MSCs. The meniscus and surrounding cartilage were evaluated at 2, 4, and 8 weeks (*n* = 5). Tendon grafts increased meniscus size irrespective of synovial MSCs. Histological scores for regenerated menisci were better in the tendon + MSC group than in the other two groups at 4 and 8 weeks. Both macroscopic and histological scores for articular cartilage were significantly better in the tendon + MSC group at 8 weeks. Implanted synovial MSCs survived around the grafted tendon and native meniscus integration site by cell tracking assays with luciferase+, LacZ+, DiI+, and/or GFP+ synovial MSCs and/or GFP+ tendons. Flow cytometric analysis showed that transplanted synovial MSCs retained their MSC properties at 7 days and host synovial tissue also contained cells with MSC characteristics. Synovial MSCs promoted meniscus regeneration augmented by autologous Achilles tendon grafts and prevented cartilage degeneration in rats. Stem Cells *2015;33:1927–1938*

## Introduction

A meniscal tear is the most prevalent injury of the knee joint [1,2], and has been identified as a strong risk factor for knee osteoarthritis [3]. At present, arthroscopic partial meniscectomy is commonly performed for meniscus injury [4], but it results in a meniscus defect that also causes the progression of cartilage degeneration [2,5]. For a meniscus substitution, meniscal allograft is one option; however, it carries the potential risk of immune response, and in the 10-year follow-up studies in second-look surgeries, symptoms showed more than a 50% failure rate [6,7]. Artificial meniscus treatments have also been reported [8,9]; however, these were often eliminated in the joint environment. Autologous tendon grafts were previously attempted for meniscus replacement [10,11], offering the advantages of safety, utility, and biological collagen properties similar to the peripheral half of the native meniscus [12], but tendon tissues were inferior to those of the meniscus due to the different types of cells present in a sheep model by Kohn et al. [10,12] and in a clinical study by Johnson and Feagin [12].

Mesenchymal stem cells (MSCs), first described in human bone marrow [13,14], are a promising cell source for regenerative medicine including meniscus regeneration. There have been a number of reports showing that the transplantation of MSCs promoted meniscus regeneration in animal models [15–17]. However, according to a clinical study recently reported by Vangsness et al. [18], intra-articular injection of bone marrow MSCs after partial meniscectomy increased meniscal volume (defined a priori by a 15% threshold) in only 24% of cases, as determined by quantitative MRI.

Clinical studies by Johnson and Feagin and Vangsness et al. suggest that it is difficult to regenerate the meniscus using only tendon grafts or only MSC transplantations in clinical situations. In comparison with bone marrow MSCs, synovial MSCs first reported by De Bari et al. [19], have the advantage of higher proliferation with similar chondrogenic potential [20,21]. The number of MSCs with characteristics of synovial MSCs increased after meniscus injury [22], and intra-articular injections of synovial MSCs enhanced meniscus regeneration in animal models [23,24]. Physiologically, synovial MSCs may have a role in meniscus healing in the natural course. In this study, we investigated whether exogenous synovial MSCs promoted meniscus regeneration augmented by autologous Achilles tendon grafts to prevent cartilage degeneration in a rat partial meniscus defect model. If the effectiveness of this method is demonstrated, and its mechanisms are elucidated, this procedure could be applied for meniscectomy patients in clinical situations.

## Materials and Methods

### Animals

Wild-type male Lewis rats (Charles River Laboratories Japan, Kanagawa, Japan) at 10–12 weeks old were used for these experiments. All animal care and experimentation were conducted in accordance with the institutional guidelines of the Animal Committee of Tokyo Medical and Dental University. To prepare MSCs for analysis of in vivo imaging, and detection of X-Gal staining and green fluorescence protein (GFP); luciferase expressing transgenic rats [25], LacZ expressing transgenic rats, and GFP expressing rats [26] (provided by Jichi Medical University, Tochigi, Japan) were also used.

### Preparation of Synovial MSCs

Synovial tissue was harvested from the intact knee joint of wild type Lewis rats (*n* = 6). Synovium was minced, digested with collagenase V for 3 hours, filtered with 70 μm cell strainer (Greiner Bio-One GmbH, Frickenhausen, Germany), and centrifuged at 1500 rpm for 5 minutes. Synovial cells were cultured in a complete culture medium; α-minimal essential medium (α-MEM/Invitrogen, Carlsbad, CA) containing 10% fetal bovine serum (FBS; Invitrogen), 100 units/ml penicillin (Invitrogen), 100 μg/ml streptomycin (Invitrogen), and 250 ng/ml amphotericin B (Invitrogen) at 37°C with 5% humidified CO_2_. After 14 days, cells were collected by trypsin, counted and preserved at −80 degrees in cell freezing medium. One million synovial MSCs at passage 3-4 were prepared in 50 μl of phosphate-buffered saline (PBS) for administration. Synovial tissue was also harvested from the intact knee joint of transgenic rats expressing luciferase (*n* = 3), LacZ (*n* = 3), or GFP (*n* = 3), and synovial MSCs were prepared in the same manner (Luc^+^ MSCs, LacZ^+^ MSCs, and GFP^+^ MSCs). For cell tracking, a fluorescent lipophilic tracer DiI (Molecular Probes, Eugene, OR) was used as described previously [27–29]. The cells were suspended at 1 million cells per milliliter in α-MEM without FBS, and DiI was added at a final concentration of 5 μl/ml. After incubation for 20 minutes at 37°, the cells were washed twice with PBS.

### Surgery

For anesthesia, isoflurane inhalation and intraperitoneal injection of tribromoethanol were performed. The Achilles tendon was harvested from the right ankle, molded into a similar size meniscus, and the tendon was immersed in the synovial MSC suspension for 10 minutes. The left knee joint was exposed with a straight skin incision, the patellar tendon was dislocated laterally, and the anterior half of the medial meniscus was resected. The prepared tendon was grafted into the meniscus defect and sutured with the joint capsule and medial collateral ligament with 6-0 nylon sutures. The residual MSCs suspension was also administrated into the knee joint after closing the patellar tendon and capsule. The rats were allowed to walk freely in their cages, and evaluated for meniscus regeneration and cartilage degeneration at 2, 4, and 8 weeks after the surgery (Tendon + MSC group; *n* = 5). The same number of rats had Achilles tendon graft surgery without synovial MSCs (Tendon group; *n* = 5) or only meniscectomy (Untreated group; *n* = 5).

### Macroscopic Observation

The tibial plateau with menisci was carefully separated from the femoral condyle. Macroscopic pictures were taken using an Olympus MVX 10 (Olympus, Tokyo, Japan), on a dedicated medical photography platform. Quantification of the size of the regenerated meniscus was performed using Axio Vision Rel software version 4.8 (Carl Zeiss, Oberkochen, Germany) to measure the ratio of the whole area of the medial meniscus including both the regenerated region and normal region, to the whole area of the medial tibial plateau [30]. Quantitative analysis of cartilage injury in the medial tibial plateau was evaluated by modified Inoue score [31].

### Histological Examination

Regenerated meniscus tissue or proximal tibia were fixed in 4% paraformaldehyde for 7 days, decalcified in 20% EDTA solution for 10 days or 21 days, then embedded in paraffin wax. The specimens were sectioned in the axial plane at 5 μm and stained with safranin-o and fast green. Histological sections were visualized using an Olympus BX 53 microscope (Olympus, Tokyo, Japan). The regenerated meniscus was evaluated using the quantitative score based on the Pauli's score (Regenerated meniscus score; Supporting Information Table 1) [32]. Cartilage degeneration of the medial tibia was evaluated with the Mankin score, on a scale of 0–14 points [33]. As a control, a normal rat at the age of 20 weeks was demonstrated for both meniscus and cartilage injury. The age was 20 weeks both in rats 8 weeks after the surgery and rats for the normal controls.

### Immunohistochemistry

Paraffin-embedded sections were deparaffinized in xylene, rehydrated through graded alcohol, and washed in PBS. Then the samples were pretreated with 0.4 mg/ml proteinase K (DAKO, Carpinteria, CA) in Tris-HCl buffer for 15 minutes at room temperature for optimal antigen retrieval. All subsequent incubations were performed in a humidified chamber. Endogenous peroxidases were quenched using 0.3% hydrogen peroxidase in methanol for 15 minutes at room temperature. Any residual enzymatic activity was removed by washing with PBS, and nonspecific antigen binding was blocked by preincubation with PBS containing 10% normal horse serum (Vector Laboratories, Burlingame, CA) for 20 minutes at 4°C. Primary antibodies (human anti-type II collagen, 1:200 dilution; Daiichi Fine Chemical, Toyama, Japan) were applied to sections and incubated at room temperature for 1 hour. After extensive washes with PBS, the sections were incubated in a secondary antibody of biotinylated horse anti-mouse IgG for type II collagen (1:200 in dilution; Vector Laboratories) for 30 minutes at room temperature. Immunostaining was detected with the Vectastain ABC reagent (Vector Laboratories) followed by diaminobenzidine staining. The sections were counterstained with hematoxylin.

### In Vivo Bioluminescent Imaging

A noninvasing bioimaging system IVIS (Xenogen, Alameda, CA) was used after the transplantation of Achilles tendon with Luc^+^ MSCs (*n* = 2). Under anesthesia with isoflurane, d-lucifein was administrated (20 μg/μl, 50 μl) into the knee joint at day 1 and weeks 2, 4, 6, 8, 10, and 12 after the surgery, and photons were detected with IVIS. The signal intensity was quantified as photon flux in units of photons per seconds in the region of interest.

### Detection of LacZ Expression

X-Gal staining was performed at 2 weeks after the transplantation of tendon with LacZ^+^ MSCs (*n* = 2). The knee specimens were fixed with a fixative solution (0.2% glutaraldehyde, 2 mM MgCl_2_, and 5 mM EDTA) in PBS for 30 minutes at room temperature and rinsed three times in PBS to wash out the fixative solution. They were treated with an X-gal staining solution (1 mg/ml 5-bromo-4-chloro-3-indolyl-β-d-galactopyranoside, 2 mM MgCl_2_, and 6 mM potassium ferrocyanide) under incubation at 37°C for 3 hours. After taking pictures of macroscopic findings, they were subsequently fixed again in 4% paraformaldehyde. The specimens were decalcified with 0.5 M EDTA (pH 7.5) for 10 days and embedded in paraffin wax, followed by sectioning and counterstaining with eosin.

### Fluorescent Macroscopic and Microscopic Examination

For the detection of GFP in the GFP^+^ tendon graft into the wild type rat, or wild type tendon graft into the GFP^+^ rat (*n* = 2, each), fluorescence images were taken using an Olympus MVX10. After macroscopic observation, SCEM (Leica Microsystems, Wetzler, Germany) was added gently into the holder. The holder was frozen in hexane chilled by dry ice and stored at −80 degree. Cryosections (10 μm) were prepared with Leica CM3050S (Leica Biosystems, Nussloch, Germany). To counter stain nuclei, hoechst dye was applied to the sections. Fluorescent images were taken using an Olympus BX 53.

### Flow Cytometry

After the regenerating meniscus was digested with collagenase in the GFP^+^ tendon graft into the wild type rat, or wild type tendon graft into the GFP^+^ rat (*n* = 2, each), cells were stained with a monoclonal antibody of APC-conjugated CD90. Propidium iodide (PI) fluorescence was measured, and a live cell gate was defined that excluded the cells positive for PI. Additional gates were defined as positive for GFP and CD90. Flow-cytometric analysis and sorting were performed on a MoFlo (Beckman Coulter, FL), and the data were analyzed using FlowJo software (Tree Star; Ashland, OR). Double positive cells were further analyzed for CD29, CD31, and CD45 (Biolegend, San Diego, CA).

### Statistical Analysis

The StatView 5.0 program (SAS Institute, Cary, NC) was used for statistical analyses. Nonrepeated measures analysis of variance was performed for analysis of the meniscus covering ratio (continuous variables). Kruskal Wallis test was performed for analysis of the regenerated meniscus score, modified Inoue score, and Mankin score (noncontinuous variables). *p* < 0.05 were considered to be statistically significant.

## Results

### Synovial MSCs Promoted Meniscus Regeneration with Grafted Tendons

**Figure 1:**
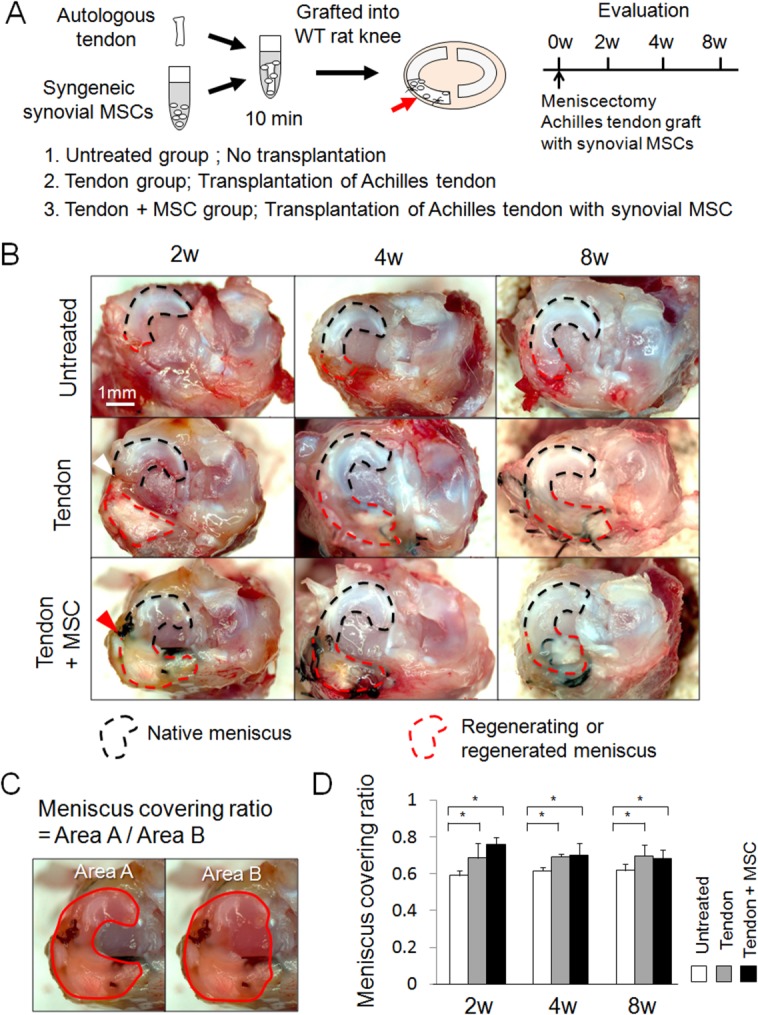
Macroscopic analyses for regenerated meniscus grafted with Achilles tendon and synovial mesenchymal stem cells (MSCs). (A): Study schema. (B): Macroscopic observation. White arrowhead indicates poor integration of the native meniscus and the grafted tendon. Red arrowhead indicates better integration of both tissues. (C): Explanation for “meniscus covering ratio,” defined as the ratio of medial meniscus area to medial plateau area. (D): Meniscus covering ratio. Bars show the mean ± SD (*n* = 5). *, *p* < 0.05 by nonrepeated measure analysis of variance. Abbreviations: MSC, mesenchymal stem cell; WT, wild type.

To determine whether synovial MSCs promoted the regeneration of meniscus by grafting of autologous Achilles tendon, we immersed the Achilles tendon in synovial MSC suspension for 10 minutes and then grafted it into the meniscus defect (Fig. 1A). Macroscopically, in the untreated group, the regenerated tissue gradually enlarged, but the size of the regenerated meniscus was still limited at 8 weeks (Fig. 1B). In the tendon group, the grafted tendon did not integrate with the native meniscus at 2 weeks (Fig. 1B, white arrowhead) and the native meniscus and regenerating tissue were distinguishable at 4 weeks and 8 weeks. In the tendon + MSC group, the grafted tendon had already integrated with the native meniscus at 2 weeks (Fig. 1B, red arrowhead), the border further matured at 4 weeks, and the native meniscus and the grafted tendon appeared to form one regenerated meniscus without identifiable borders at 8 weeks (Fig. 1B). The meniscus coverage ratio (Fig. 1C) was smaller in the untreated group than in the other two groups throughout the study (Fig. 1D).

**Figure 2:**
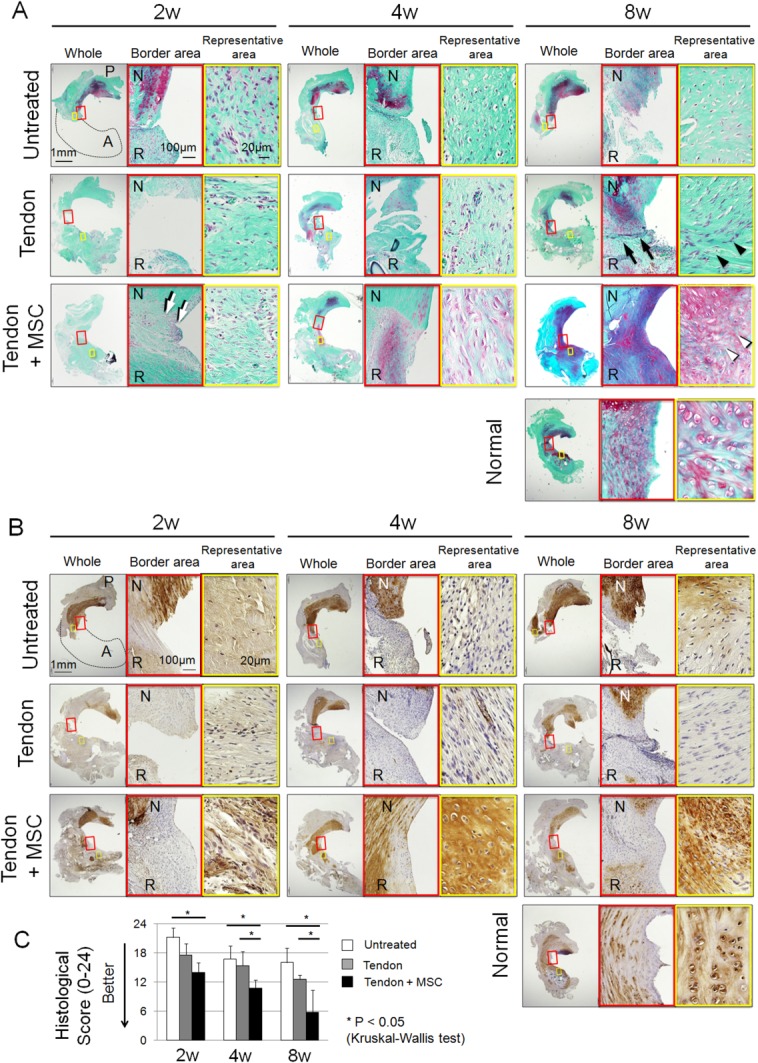
Histological analyses for regenerated meniscus. (A): Sections stained with safranin-o. The left panels in each time period show the whole medial meniscus. The red square shows border area between the native meniscus and the grafted tendon. The yellow square shows representative area during the regenerating process. One 20-week old normal rat was used as a control. (B): Sections immunostained with type II collagen. The red square shows border area between the native meniscus and the grafted tendon. The yellow square shows representative area during the regenerating process. One 20-week old normal rat was used as a control. (C): Pauli's histological score for regenerated meniscus. Bars show the mean ± SD (*n* = 5). *, *p* < 0.05 by Kruskal–Wallis test. Abbreviations: A; anterior, P; posterior, N; native meniscus, R; regenerated meniscus; MSC, mesenchymal stem cell.

Histologically, in the untreated group, only coarse synovial tissue was observed at the end of the resected meniscus at 2 weeks, and remained virtually unchanged at 4 and 8 weeks (Fig. 2A). In the tendon group, the native meniscus and the grafted tendon formed a C-shaped tissue but they were still separated completely at 2 weeks and partially at 4 weeks. The native meniscus and the grafted tendon almost completely integrated at 8 weeks, but the border was still identifiable (Fig. 2A, black arrows). The morphology of the cells in the regenerating meniscus was distinct from meniscal cells in the normal meniscus (Fig. 2A, black arrowheads). In the tendon + MSC group, the border was filled with synovial tissue at 2 weeks (Fig. 2A, white arrows), and it appeared smoother and matrix at the border was stained partially red indicating the synthesis of proteoglycans at 4 weeks, and it was stained red equally without an identifiable border, indicating further maturation at 8 weeks. The morphology of the cells in the regenerated meniscus was similar to that of meniscal cells in the normal meniscus (Fig. 2A, white arrowheads).

Type II collagen expression in the areas of regeneration was hardly observed throughout the study in both the untreated and tendon groups, contrarily, it was clearly observed at 4 and 8 weeks in the tendon + MSC group (Fig. 2B).

Histological scores for regenerated menisci were better in the tendon + MSC group than in the untreated group at 2, 4, and 8 weeks, and were better than the tendon group at 4 and 8 weeks (Fig. 2C).

### Regenerated Meniscus by Achilles Tendon Graft with Synovial MSCs Prevented Cartilage Degeneration

**Figure 3:**
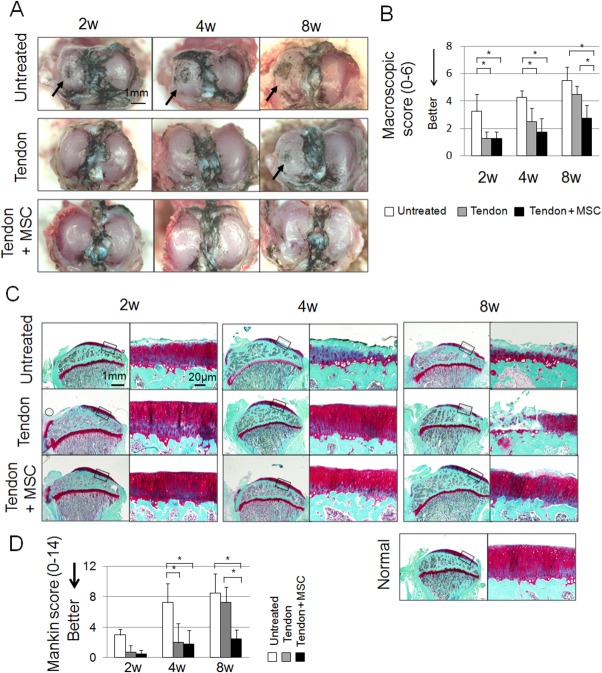
Macroscopic and histological analyses for articular cartilage at the medial tibial plateau. (A): Macroscopic features. Cartilage surface was stained with India ink. Arrows indicate cartilage erosion. (B): Modified Inoue's score. Bars show the mean ± SD (*n* = 5). *, *p* < 0.05 by Kruskal–Wallis test. (C): Sagittal sections stained with safranin-o. 20-weeks-old normal rat was also used as a control. (D): Mankin scores. Bars show the mean ± SD (*n* = 5). *, *p* < 0.05 by Kruskal–Wallis test. Abbreviation: MSC, mesenchymal stem cell.

We then evaluated the chondroprotective effect of the regenerated meniscus. Macroscopically, in the untreated group, cartilage erosion was already detected at 2 weeks and progressed over 4 and 8 weeks (Fig. 3A, arrows). In the tendon group, cartilage erosion was mild at 4 weeks and increased at 8 weeks. In the tendon + MSC group, the cartilage surface remained better preserved, and the macroscopic score at 8 weeks was better than the other 2 groups (Fig. 3B).

Histological examination yielded results similar to the macroscopic findings. Cartilage degeneration progressed in the untreated group and the tendon group over 8 weeks, whereas cartilage was considerably more preserved in the tendon + MSC group (Fig. 3C). Mankin score in the tendon + MSC group at 8 weeks was significantly better than other groups (Fig. 3D).

### Transplanted MSCs Survived Around the Grafted Tendon Including at the Integration Site

**Figure 4:**
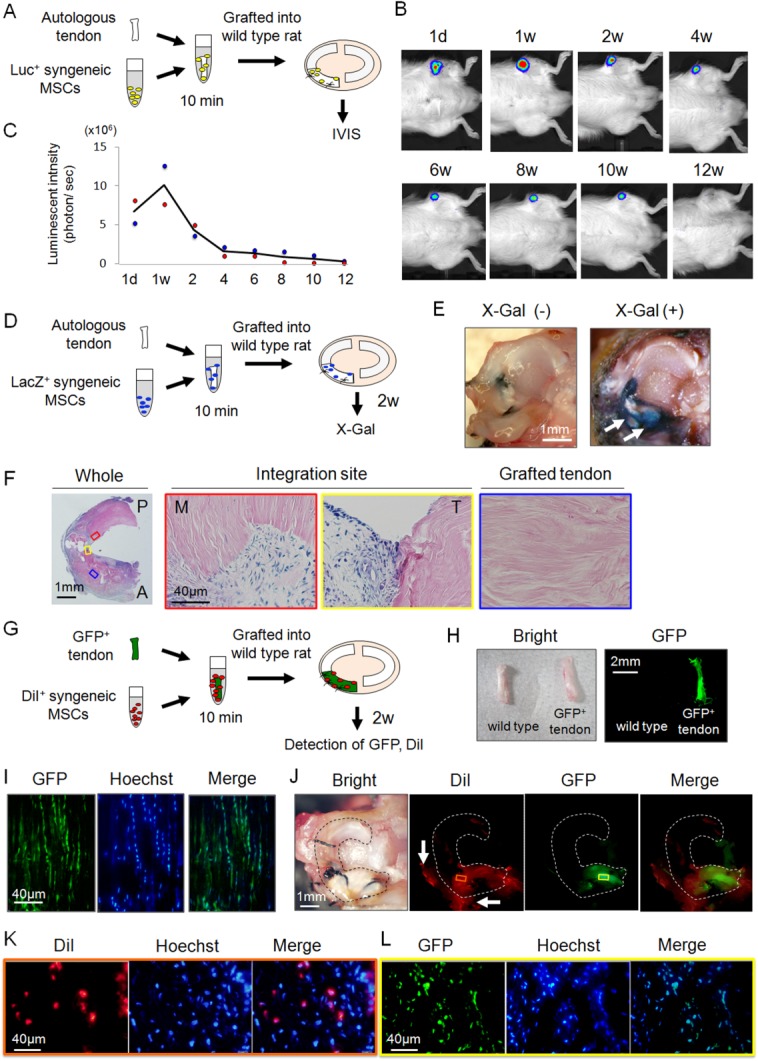
Detection of transplanted synovial mesenchymal stem cells (MSCs) and grafted tendon. (A): Schematic representation of the bioluminescent in vivo imaging analysis (IVIS). (B): Detection of photons from synovial MSCs derived from a luciferase expressing transgenic rat. (C): Sequential quantification of luminescence intensity. Raw data are plotted and the averaged values are shown as a line (*n* = 2). (D): Schematic representation of the detection of transplanted MSCs derived from a LacZ expressing transgenic rat (*n* = 2). (E): Macroscopic features after X-Gal staining for tibial plateau with medial meniscus. White arrows indicate the LacZ positive area. (F): Histological analysis of LacZ positive cells after X-Gal staining. The red and yellow squares show the integration site of native meniscus side and grafted tendon side respectively. The blue square shows the inner part of the grafted tendon. (G): Schematic representation of analyses of regenerated meniscus grafted with green fluorescence protein (GFP^+^) Achilles tendon with DiI^+^ MSCs (*n* = 2). (H): Macroscopic features of GFP^+^ Achilles tendon. (I): Histology of GFP^+^ tendon. (J): Macroscopic images of regenerated meniscus. The red or yellow squares show the site of histological analysis for DiI or GFP respectively. White arrows indicate the synovium which covered the grafted tendon. (K): Histological analysis of DiI^+^ MSCs in the regenerated meniscus. (L): Histological analysis of GFP^+^ tendon cells in the regenerated meniscus. Abbreviations: GFP, green fluorescence protein; IVIS, in vivo imaging analysis; MSC, mesenchymal stem cell.

To examine cell migration and survival, we used Luc^+^ MSCs and evaluated photons through an IVIS system (Fig. 4A). Luc^+^ MSCs were detected only around the knee joint, and were undetectable elsewhere (Fig. 4B). MSC-derived photons increased at 1 week, then decreased thereafter, remaining detectable until 10 weeks (Fig. 4C).

We then evaluated the distribution of transplanted LacZ^+^ MSCs in the knee joint (Fig. 4D). At 2 weeks, LacZ^+^ areas were detected around the grafted tendon macroscopically (Fig. 4E, white arrows). According to histological observations of horizontal sections for the mid portion of the tendon, LacZ^+^ MSCs were confirmed in the integration site, but not within the grafted tendon (Fig. 4F).

Next, to analyze the relationship of grafted tendon cells and transplanted MSCs (Fig. 4G), Achilles tendons from syngeneic GFP^+^ rats (Fig. 4H, 4I) were grafted and synovial MSCs labeled with DiI were transplanted. At 2 weeks, GFP^+^ areas were macroscopically confirmed only in the grafted tendon, and DiI^+^ areas were detected around the GFP^+^ tendon, including the integration site and anterior synovium (Fig. 4J). Histologically, DiI^+^ cells were confirmed in the synovial tissue (Fig. 4K) and GFP^+^ cells were detected in the grafted tendon (Fig. 4L).

### Transplanted Synovial MSCs Retained MSC Properties and Host Synovial Tissue Also Contained Cells with MSC Characteristics

**Figure 5:**
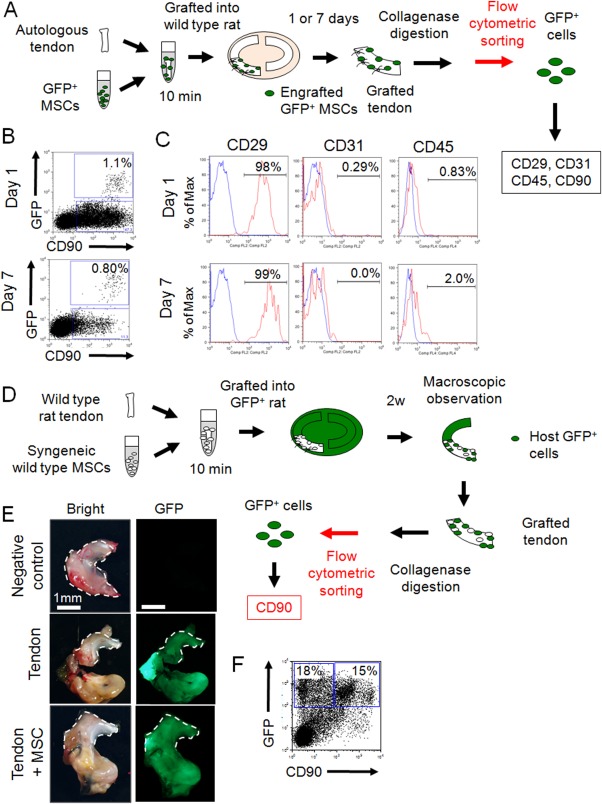
Analyses of mesenchymal stem cell (MSC) properties after engraftment and contribution of host knee tissue. (A): Schematic representation of the flow cytomeric assay of green fluorescence protein (GFP^+^) MSCs around the regenerating meniscus (*n* = 2). (B): Representative flow cytometric profiles of regenerating meniscus for GFP^+^ and CD90^+^ cells. (C): Expression of the indicated markers in CD90 and GFP double positive MSCs. Shown is the percentage of cells that express the antigen (red line) versus a matched isotype control (blue line). (D): Schematic representation of the analysis of the contribution of host knee tissue (*n* = 2). (E): Macroscopic analysis for GFP detection of grafted WT tendon into GFP^+^ rat knee. White dotted lines indicate the native meniscus. (F): Representative flow cytometric profiles of regenerating meniscus for GFP^+^ and CD90^+^ cells. Abbreviations: GFP, green fluorescence protein; MSC, mesenchymal stem cell; WT, wild type.

Next, GFP^+^ MSCs were transplanted, and GFP^+^ MSCs that would contribute to meniscus regeneration were analyzed using flow cytometry (Fig. 5A). At Day 1, GFP^+^ cells comprised 1.1% of the regenerating meniscus, and nearly all of these GFP^+^ cells were positive for CD90 (Fig. 5B). The cells were also positive for CD29, while negative for CD31 and CD45 (Fig. 5C) and this expression patterns was the same at Day 7 (Fig. 5B, 5C).

Finally, to elucidate the contribution of host knee tissues for meniscus regeneration, Achilles tendons were grafted, and MSCs were transplanted to GFP^+^ rat knees (Fig. 5D). At 2 weeks, GFP^+^ synovial tissue covered not only the grafted tendon but also the native meniscus irrespective of MSC transplantation (Fig. 5E). Flow cytometric analysis revealed that approximately half of these GFP^+^ cells covering the grafted tendon were CD90^+^ cells (Fig. 5F).

## Discussion

We successfully established a novel procedure for meniscus regeneration by combining autologous tendon grafts and synovial MSCs. Tendon grafts served as scaffolds to cover the meniscus defect just after the surgery, which is the similar to results observed when bone morphogenetic protein 7 (BMP-7) treated tendons were implanted in a similar rat model [30].

Many scaffolds, including collagen implants and urethane implants, have been utilized for meniscus regeneration [34,35], but these materials were usually eliminated in the joint environment by possible rejection or excessive stress due to their mismatched size, comparative to the native meniscus. Alternatively, an autologous tendon graft is a safe material commonly used for ligament reconstruction [36], and easy to handle to fit the length and size suitable for the meniscus. In this study, the grafted tendon was able to survive in the knee joint at least 8 weeks without elimination, which indicates that tendon grafts could be one suitable scaffold for meniscus regeneration.

In our previous *in vivo* analyses, after a GFP+ synovial MSC suspension was put in place for 10 minutes, the cells could be observed in cartilage defects [37] and in torn menisci [38] in pig models. Our analyses showed that approximately 60% of synovial MSCs attached to the cartilage defect 10 minutes after MSC suspension was placed on the cartilage defect [39]. Placing synovial MSC suspension on the cartilage and meniscus lesions for 10 minutes allowed synovial MSCs to adhere with low invasion. In our previous rat study without scaffolds, photons of Luc^+^ MSCs rapidly decreased after intra-articular administration and disappeared within 1 week, in a similar rat meniscus defect model [40]. In this study, we immersed the harvested tendon in synovial MSC suspension before transplantation, and the remaining suspension was also administered into the joint after transplantation. Photons of Luc^+^ MSCs could be detected for up to 10 weeks, using the same number of cells. Although we did not quantify the number of cells adhered to the tendon, our current procedure enhanced the adherence and maintenance of MSCs to the tendon specifically rather than the more diffuse administration into the entire knee joint.

Synovial MSCs were able to bind to the excised tendon within 10 minutes of immersion. We previously examined the relationship between integrin expression and attachment to type 1 collagen-coated dishes in human synovial MSCs [41]. Synovial MSCs expressed integrin α5, β1 highly, integrin α2, α3 modestly, and integrin α4 at a low level. The attachment of synovial MSCs on the type 1 collagen-coated dish was diminished by the neutralizing antibodies for integrin α3 and β1. The tendon consists of primarily type 1 collagen. These indicate that synovial MSCs attach to the tendon through α3 and β1 integrins.

We did not test MSC transplantation alone in these studies. Our previous study with MSC administration alone showed better meniscus regeneration 2 weeks after the surgery than the control group, but the size of the regenerated meniscus was much smaller than the native meniscus [23]. In this study, using both tendon grafts and MSCs were more effective not only for MSC survival, but also to obtain larger regenerated menisci from an earlier stage.

Though the definition of MSCs is still controversial, a minimum criterion for MSCs was advocated in 2006, in which MSCs were defined by adherence to plastic, colony formation, trilineage differentiation, and surface markers [42]. We previously reported that cells derived from rat synovium formed colonies, differentiated into chondrocytes and adipocytes, and were calcified when cultured in the appropriate differentiation medium, and were positive for CD90 and CD29, and negative for CD11b, CD31, CD34, and CD45 [21,23,43]. Therefore, we defined the cells used in this study as MSCs.

Generally, endogenous stem cells are recruited to the injured site [44] and administered stem cells are likely to adhere to the injured tissue [23]. Using LacZ^+^ MSCs and DiI^+^ MSCs, we confirmed that these cells remained around the meniscus defect, especially within the integration site. Macroscopic and histological observations showed transplanted synovial MSCs distributed on the surface of the grafted tendon, but they were not detected within the grafted tendon. However, safranin-o and type II collagen staining in the grafted tendon increased. These findings indicate that transplanted MSCs promoted not only the junction between the native meniscus and the grafted tendon but also the remodeling of the grafted tendon itself.

Although an increasing number of MSC studies are emerging, it still remains unclear whether these cells retain their MSC properties after engraftment. We successfully sorted implanted GFP^+^ MSCs by flow cytometry, and we determined most GFP^+^ MSCs retained their MSC properties 1 day and 7 days after transplantation. Surviving synovial MSCs did not fully differentiate into the regenerated meniscus cells within 7 days.

In the process of meniscus regeneration after the transplantation of tendon grafts, synovial coverage from the host knee is a critical factor [30]. When tendon was grafted alone, synovial coverage of the integration site was observed at 4 weeks after the surgery. However, the native meniscus and regenerating tissue were distinguishable even at 8 weeks histologically. On the other hand, when synovial MSCs and tendon were applied together, the integration was obtained as early as 2 weeks after the surgery, and the native meniscus and the grafted tendon appeared to form one regenerated meniscus without an identifiable border at 8 weeks, histologically. In the analysis of wild type rat tendon grafts in the meniscus defects of GFP^+^ rats, grafted tendons were covered by GFP^+^ synovial tissue. These results indicated native synovial MSCs also promoted the coverage of grafted tendon by host knee synovial tissue, and contributed to healing of the tendon graft and the native meniscus.

Important observations were made of the regenerative contributions of the grafted synovial MSCs and the native synovium over time. In vivo imaging analysis revealed that administrated MSCs gradually decreased 2 weeks after surgery. We recently examined whether the transplantation of synovial MSCs promoted healing after meniscal repair of an extended longitudinal tear of the avascular area in a microminipig model. Synovial tissue showed better coverage along the superficial layer from the outer zone into the lesion of the meniscus even at 2 weeks after MSC transplantation and promoted healing after meniscal repair thereafter [38]. These findings suggest that the grafted synovial MSCs induce synovial coverage at the early phase, which helps to promote meniscus regeneration.

One of the mechanisms of stem cell therapy is the production of trophic factors [45]. From our previous studies, synovial MSCs express BMPs after migration within the knee joint [29,40], which are critical for the differentiation of chondrocytes or cartilage matrix synthesis [46]. In addition, we also reported that BMP-7 promoted meniscus regeneration by tendon grafting [30]. These findings indicate that the administered MSCs secreted cytokines including BMPs, which promoted the remodeling of the grafted tendon.

In this study, we used young rats at 10–12 weeks and our follow-up period was just 8 weeks after the surgery. The healing potential of rats is higher than that of humans in the meniscus [23], bone [47], and tendon [48]. Furthermore, the healing potential of younger rats is higher than that of older rats [49]. We should take these differences in species and age into consideration when considering the application of these data in young rats to humans.

For the meniscus defect model in rats, we transected the anterior half of the meniscus. The transaction of the anterior half of the meniscus is simpler to perform that the transaction of the posterior half of the meniscus, therefore, this model has a high reproducibility and is less invasive [23,43,50]. However, rats naturally ambulate with their knee joint more flexed than humans, which causes less loading on the anterior part of the knee joint, and it may influences meniscus regeneration. Further study is necessary to clarify the effectiveness of this procedure for the posterior part of the meniscus.

For the regenerated menisci, we did not perform biomechanical analysis; therefore, we cannot conclusively confirm that the regenerated meniscus had the same biomechanical properties of the normal meniscus. This was in part due to the inherent difficulties of quantifying the compressive modulus of complex three-dimensional geometries and stress loads within and around the transition zone of the regenerated portion of the meniscus and its adjacent native meniscus tissue in rats. Although, the macroscopic findings and histological morphologies of the regenerated meniscus and its union with normal tissue were close to that of the native undamaged meniscus, and cartilage degeneration was significantly delayed in the tendon + MSC group, in this animal model which allowed for free motion and loading of the knee. These findings suggest that the biomechanical properties of this regenerated tissue were closer to that of the native rat meniscus than the tendon group and the untreated group. To overcome testing difficulties attributed to the small size of rat menisci, we plan to examine whether synovial MSCs can promote improved biomechanical properties of regenerated menisci augmented by an autologous tendon graft in microminipigs in our future work. We recently reported that the tensile strength to failure of the sutured meniscus increased after synovial MSC transplantation in microminipigs [38].

**Figure 6:**
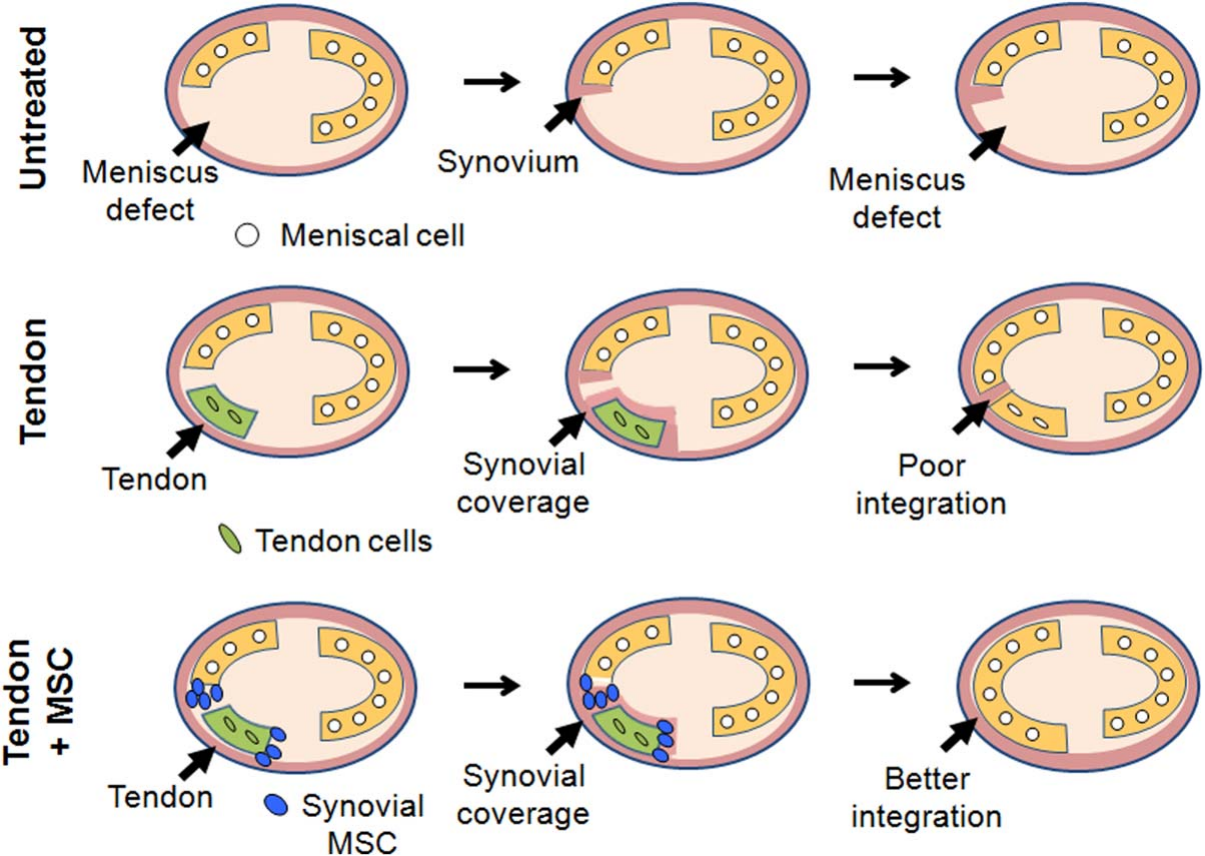
Mechanism of meniscus regeneration. Grafted tendon, transplanted synovial mesenchymal stem cells (MSCs), and host knee synovial tissue contributed to meniscus regeneration in these experiments. Abbreviation: MSC, mesenchymal stem cell.

We summarized the mechanism of meniscus regeneration in this study (Fig. 6). When meniscus defects were not treated, only a small amount of synovial tissue proliferated at the end of the native meniscus, but this synovial tissue had no effect on preventing cartilage degeneration. When the tendon was grafted to the meniscus defect, it acted as a scaffold for meniscus regeneration from the very early stages of healing. However, integration between the tendon and the native meniscus was poor and lacked the ability to restore function. When the tendon with synovial MSCs was grafted into a meniscal defect, the tendon scaffold provided a vehicle for the MSCs introduction and survival in the joint space, and these synovial MSCs improved the healing of the grafted tendon and native meniscus, in part by promoting synovial coverage at the integration site. Regenerated menisci in the tendon + MSC group attained morphological and functional characteristics similar to that of the native meniscus.

## Conclusion

Synovial MSCs promoted meniscus regeneration augmented by an autologous Achilles tendon graft, and prevented cartilage degeneration in a rat partial meniscus defect model.
