# Supplementary material for: Synovial Mesenchymal Stem Cells Promote Meniscus Regeneration Augmented by an Autologous Achilles Tendon Graft in a Rat Partial Meniscus Defect Model
Source: Stem Cells. 2015 May 21;33(6):1927–38. doi: 10.1002/stem.2030 (PMC4497612; doi:10.1002/stem.2030)
Supplement: Supplementary file 1 — Supporting Information [file stem0033-1927-sd1.docx]

Supplementary table 1

Histological scoring for regenerated meniscus

**I. Size**

0; Large

1; Moderate

2; Small

3; Not observed

**II. Morphology of regenerated meniscus**

0; C-shaped like normal meniscus

1; Gradual C-shape

2; Distinct shape from meniscus

3; No appearance of meniscus

**III. Surface integrity**

0; Smooth

1; Slight fibrillation or slightly undulating

2; Moderate fibrillation or markedly undulating

3; Severe fibrillation or disruption

**IV. Integration with native meniscus**

0; Complete integration without detectable border

1; Incomplete integration with detectable border

2; Separated with narrow space

3; Far from native meniscus

**V. Cellularity of meniscal cells**

0; Normal cell distribution

1; Almost normal cell distribution

2; Hypercellularity or hypocellularity

3; No meniscal cells

**VI. Cell morphology**

0; Similar to normal chondrocyte

1; Round shape, but small or hyperplastic

2; Mix of round cells and other shapes of cells

3; No chondrocyte shaped cells

**VII. Collagen fiber organization**

0; Collagen fibers well organized, no separations or tears

1; Collagen fibers moderately well organized, slight separations or tears

2; Collagen fibers unorganized, moderate separations or tears

3; Collagen fibers unorganized, severe separations or tears

**VIII. Matrix staining**

0; Well stained like normal meniscus

1; Moderately stained

2; Slightly stained

3; No stain
